# ROS‐Responsive Hydrogel for Localized Delivery of Nampt and Stat3 Inhibitors Exhibits Synergistic Antitumor Effects in Colorectal Cancer Through Ferroptosis Induction and Immune Microenvironment Remodeling

**DOI:** 10.1002/advs.202506599

**Published:** 2025-06-10

**Authors:** Chenyang Ye, Mi Mi, Saimeng Shi, Lina Qi, Shanshan Weng, Lu Wang, Yier Lu, Chao Chen, Yinuo Tan, Mengyuan Yang, Cheng Guo, Rui Bai, Xuefeng Fang, Ji Wang, Ying Yuan

**Affiliations:** ^1^ Department of Medical Oncology Cancer Institute Key Laboratory of Cancer Prevention and Intervention School of Medicine Ministry of Education The Second Affiliated Hospital of Zhejiang University Hangzhou Zhejiang 310009 China; ^2^ Center for Plastic & Reconstructive Surgery Department of Plastic &Reconstructive Surgery Zhejiang Provincial People's Hospital (Affiliated People's Hospital Hangzhou Medical College) Hangzhou 310014 P. R. China; ^3^ Department of Colorectal Surgery and Cancer Institute (Key Laboratory of Cancer Prevention and Intervention Key Laboratory of Molecular Biology in Medical Sciences China National Ministry of Education; Zhejiang Province China) The Second Affiliated Hospital of Zhejiang University School of Medicine Hangzhou 310058 China; ^4^ Zhejiang Provincial Clinical Research Center for CANCER Hangzhou Zhejiang 310009 China

**Keywords:** colorectal cancer, ferroptosis, FK866, reactive oxygen species (ROS)‐responsive hydrogel, tumor immune microenvironment

## Abstract

Targeting Nampt to modulate NAD^+^ metabolism and exert antitumor effects has become a research hotspot in the field of cancer metabolism. But early clinical trials have only achieved modest results, primarily due to the need for improved efficacy and the occurrence of severe systemic adverse effects. Therefore, enhancing antitumor efficacy and reducing the adverse effects of Nampt inhibitors are urgent challenges. The research reveals that the Nampt inhibitor FK866 can induce ferroptosis in colorectal cancer cells via the NAD^+^/Stat3/Gpx4 signaling axis. Furthermore, the combination of FK866 and the Stat3 inhibitor C188‐9 demonstrates a strong synergistic antitumor effect. Importantly, a reactive oxygen species (ROS)‐responsive hydrogel that encapsulates FK866 and C188‐9 for in situ drug delivery, effectively reducing systemic side effects, is developed. Intriguingly, mass cytometry time‐of‐flight (CyTOF) analysis indicates that the combined treatment with FK866 and C188‐9 exerts antitumor effects by increasing the infiltration of CD8^+^ T cells and neutrophils into the tumor, as well as enhancing the expression of immune‐regulatory molecules, including IFN‐γ, IL‐10, and perforin. Thus, this localized treatment not only minimizes systemic adverse effects, but also markedly amplifies antitumor efficacy through the modulation of both tumor cells and the tumor immune microenvironment, representing a promising antitumor treatment strategy.

## Graphical abstract

In this study, ferroptosis in colorectal cancer cells is induced by FK866 through the NAD⁺/STAT3/GPX4 pathway. To minimize systemic toxicity, a reactive oxygen species (ROS)‐responsive hydrogel is developed to co‐deliver FK866 and C188‐9, resulting in synergistic and localized antitumor effects.

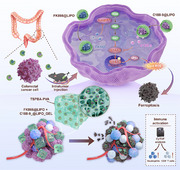

## 1 Introduction

Colorectal cancer (CRC) is a common malignancy with the second highest incidence and fourth highest mortality rate in China.^[^ ^1^ ^]^ It is estimated that 70–80% of advanced colorectal cancer cases are unresectable, with a 5‐year overall survival (OS) rate of less than 10%.^[^ ^2^ ^]^ For unresectable metastatic CRC, the primary treatment strategy is systemic therapy, encompassing chemotherapy, targeted biologic therapy, and immunotherapy.^[^ ^2^ ^]^ However, the effectiveness of systemic therapy is limited, necessitating the exploration of new therapeutic targets.

The metabolic profile of cancer cells is distinct from that of normal cells.^[^ ^3^, ^4^ ^]^ Our group^[^ ^5^, ^6^ ^]^ and others^[^ ^7^ ^]^ revealed that metabolic alterations in cancer cells contribute to cell proliferation, migration, invasion, and so on. Strategies targeting the intrinsic metabolism of cancer cells have garnered increasing attention in recent years.^[^ ^8^ ^]^ Nicotinamide adenine dinucleotide (NAD^+^) is a key coenzyme in bioenergetic processes that directly and indirectly impacts metabolic pathways and cellular functions. In mammals, the NAD^+^ salvage pathway, primarily regulated by intracellular nicotinamide phosphoribosyl transferase (Nampt), is the main source of NAD^+^.^[^ ^9^ ^]^ While Nampt is biologically essential, its overexpression is linked to tumorigenesis.^[^ ^10^ ^]^ Dysregulation of Nampt and the resulting changes in NAD^+^ levels have been observed in various cancers, making Nampt a compelling target for anticancer therapy.

Although the Nampt inhibitor FK866 has shown impressive anticancer efficacy in preclinical studies,^[^ ^6^, ^11^, ^12^, ^13^ ^]^ its clinical trial results have not met expectations. This discrepancy is largely due to suboptimal efficacy and the emergence of severe systemic adverse reactions (mainly hematological toxicity and gastrointestinal toxicities).^[^ ^14^, ^15^ ^]^ Therefore, new therapeutic strategies are urgently needed to enhance the anticancer efficacy of Nampt‐targeting therapies and reduce systemic adverse effects. To enhance the anticancer efficacy of Nampt‐targeting therapies, a thorough investigation into the underlying molecular and cellular mechanisms is essential. This will uncover new therapeutic targets and facilitate the development of more effective combination treatments. To reduce systemic adverse effects, localized drug delivery is preferred. Hydrogel is an ideal drug delivery system for in situ cancer treatment, effectively minimizing systemic exposure and side effects on normal tissues.^[^ ^16^ ^]^ Additionally, the unique features of the tumor microenvironment (TME) can be leveraged to design tumor‐specific responsive hydrogels.^[^ ^17^ ^]^ Characteristics such as elevated reactive oxygen species (ROS) levels and low pH have been demonstrated—by our group^[^ ^18^ ^]^ and others^[^ ^19^, ^20^, ^21^, ^22^ ^]^—to effectively induce hydrogel degradation at tumor sites.

**Figure 1:**
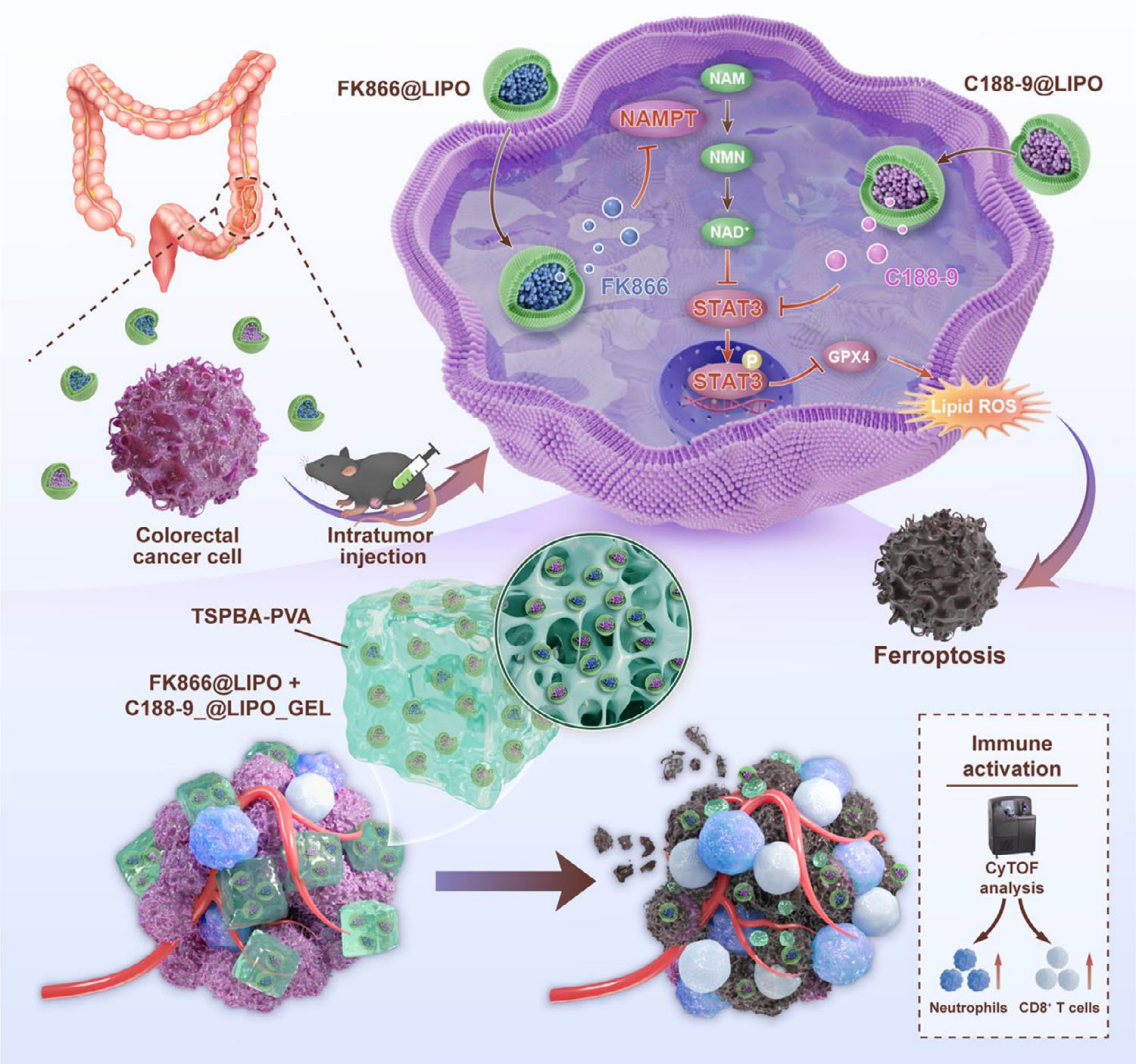
The Nampt inhibitor FK866 induces ferroptosis in CRC cells through NAD^+^/Stat3/Gpx4 signaling. A hydrogel that is responsive to ROS has been constructed for local CRC treatment. Co‐administration of the Nampt inhibitor FK866 and the Stat3 inhibitor C188‐9 elicited synergistic antitumor effects and enhanced antitumor immune responses.

In this study, we demonstrated that FK866 induces ferroptosis in CRC cells via the NAD^+^/Stat3/Gpx4 axis. A ROS‐responsive hydrogel was developed by crosslinking polyvinyl alcohol with N^1^‐(4‐boronobenzyl)‐N^3^‐(4‐boronophenyl)‐N^1^,N^1^,N^3^,N^3^‐tetramethylpropane‐1,3‐diaminium for localized drug delivery. Remarkably, combining FK866 with the Stat3 inhibitor C188‐9 showed strong synergistic anticancer effects both in vitro and in vivo. More importantly, cytometry by time‐of‐flight (CyTOF) revealed that this combination therapy reshapes the tumor immune microenvironment at single‐cell resolution, underscoring its role in enhancing anticancer efficacy. Our findings suggest that using ROS‐responsive hydrogels to deliver FK866 and C188‐9 not only significantly enhances antitumor efficacy by modulating both tumor cells and the tumor immune microenvironment, but also reduces systemic adverse effects (**Figure** **1**).

## 2 Results

### 2.1 Nampt Is Highly Expressed and Correlated with Ferroptosis in CRC

**Figure 2:**
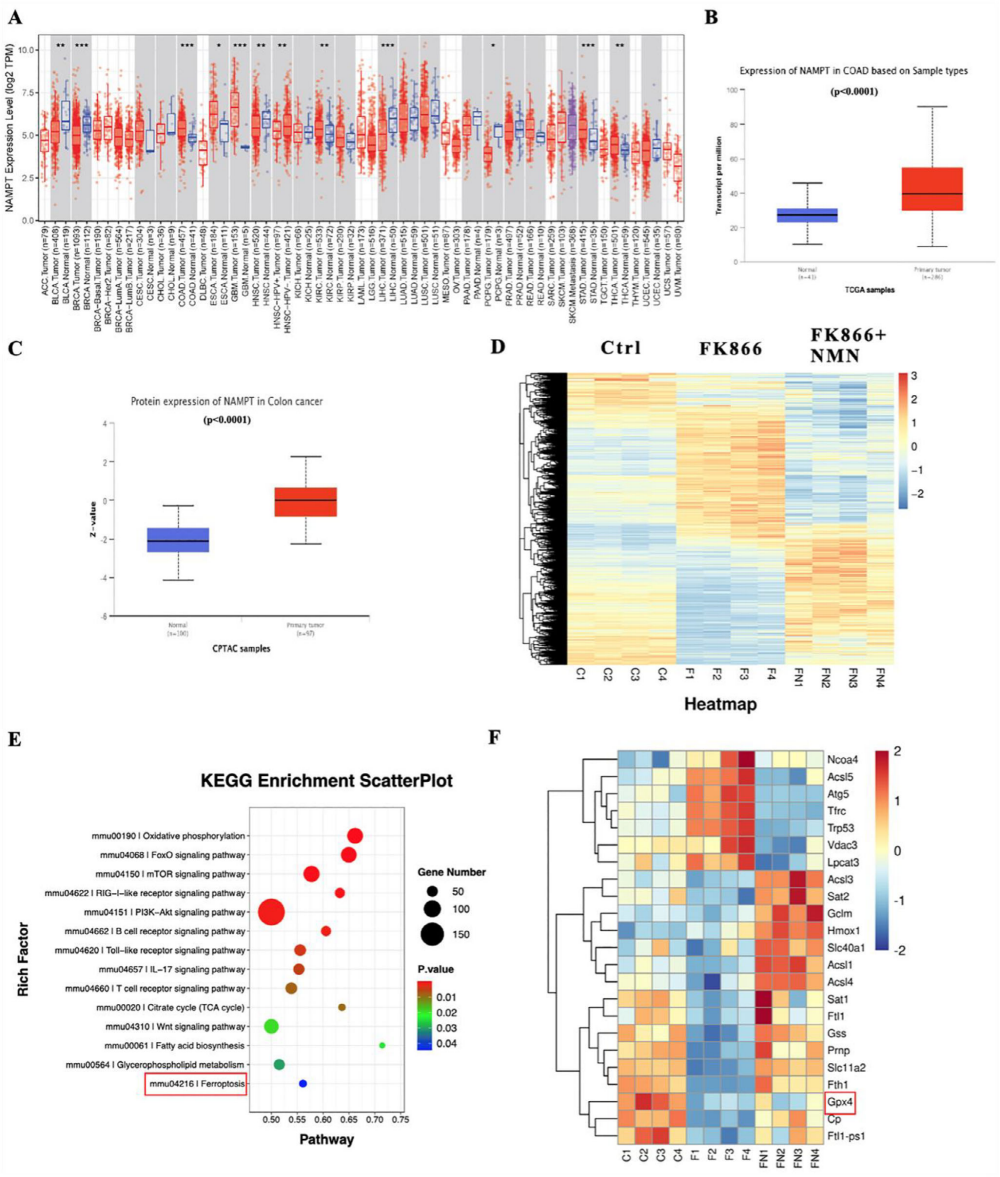
Expression of Nampt in colorectal cancer and its association with ferroptosis. A) Expression of Nampt in different tumors in The Cancer Genome Atlas (TCGA). B) Comparison of the expression of the Nampt gene in COAD between normal tissues and primary tumors based on the TCGA dataset. A total of 41 normal tissues and 286 primary tumor tissues were included in the analysis. C) Protein expression of Nampt in colon cancer tissues compared with that in primary tumor tissues based on the Clinical Proteomic Tumor Analysis Consortium (CPTAC) dataset. A total of 100 normal tissues and 97 primary tumor tissues were included in the analysis. D) Groups of RNA‐seq experiments (*n* = 4). E) KEGG results based on RNA‐seq in MC38 cells (treated with PBS vs. FK866 vs. FK866+NMN) (*n* = 4). F) Differential expression of ferroptosis‐related genes (*n* = 4). *< 0.05, **< 0.01, ***< 0.001.

Based on TCGA data, we found that Nampt was highly expressed in multiple cancers, including CRC, bladder cancer, and breast cancer (**Figure** **2**A). The expression of Nampt at both the mRNA and protein levels was greater in colon cancer tissues than in adjacent normal tissues (Figure 2B,C). To identify novel pathways related to Nampt and NAD^+^ metabolism, we conducted RNA sequencing of the CRC cell line MC38 treated with PBS, FK866 (a Nampt inhibitor), or FK866 + NMN (the downstream intermediate of Nampt) (Figure 2D). Interestingly, KEGG enrichment analysis of the RNA sequencing data indicated that ferroptosis was significantly regulated (Figure 2E). Furthermore, compared with those in the control group, the expression of ferroptosis‐related genes (including Gpx4) was differentially regulated upon FK866 treatment and could be rescued by the addition of NMN (Figure 2F). These results suggest that Nampt‐mediated NAD^+^ metabolism may influence CRC progression through ferroptosis.

### 2.2 FK866 Induces Ferroptosis in CRC Cells

**Figure 3:**
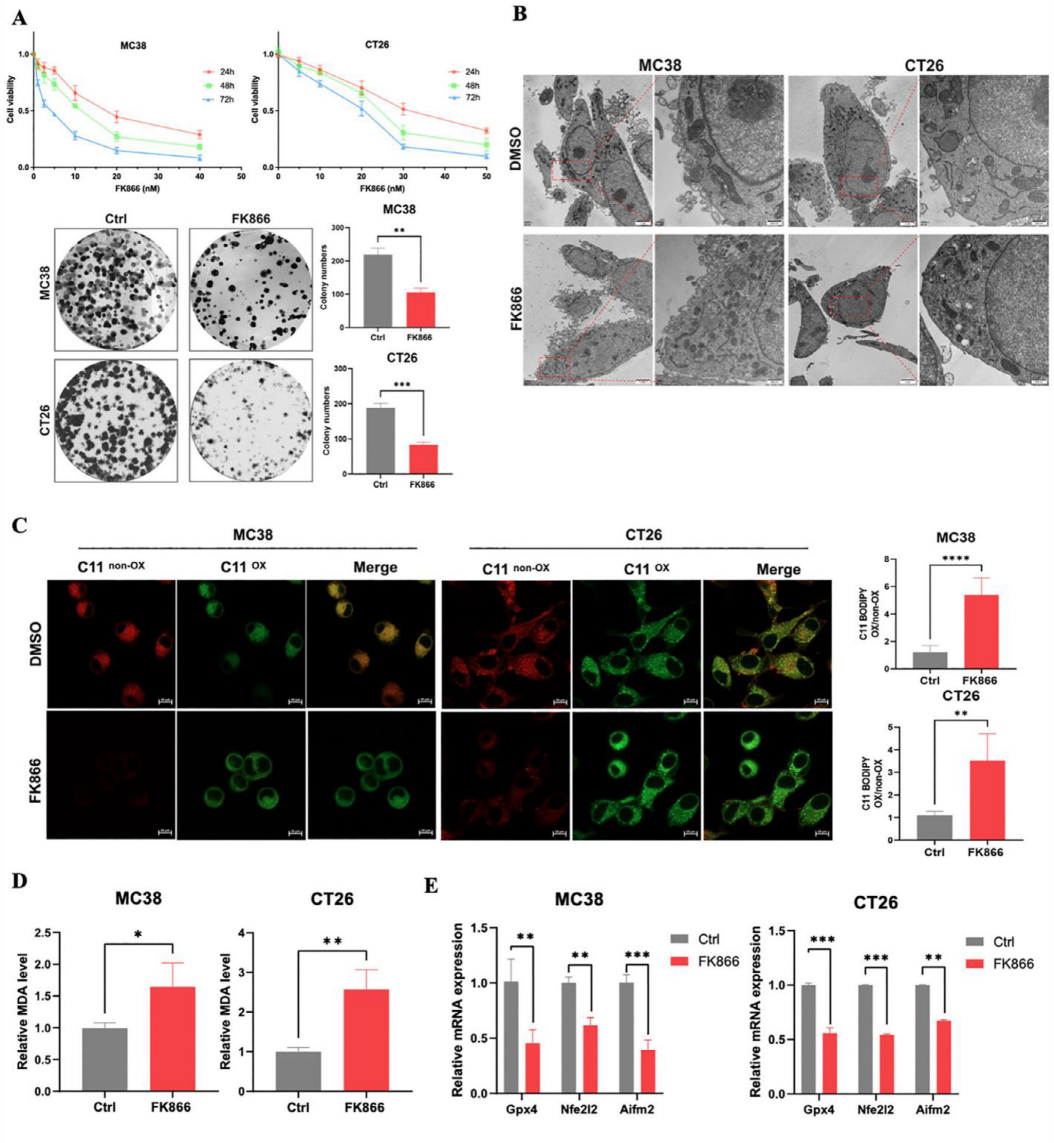
FK866 induces CRC cell death and the production of ROS and lipid ROS. A) Cell viability and colony formation assays of MC38 and CT26 cells treated with FK866 (5 nm MC38 or 10 nm CT26) (*n* = 3). B) TEM images of MC38 and CT26 cells treated with DMSO or FK866 (5 nm MC38 or 10 nm CT26) for 48 h. The scale bar is 2 µm (in the enlarged image, the scale bar is 500 nm). This experiment was conducted in triplicate. C) The relative lipid ROS levels of MC38 and CT26 cells were assayed via C11‐BODIPY fluorescence after treatment with FK866 (5 nm MC38 or 10 nm CT26) for 48 h (*n* = 3). D) The relative MDA levels in MC38 and CT26 cells after treatment with FK866 for 48 h (5 nm MC38 or 10 nm CT26) (*n* = 3). E) The relative mRNA expression of redox‐related genes (Gpx4, Nfe2l2, and Aifm2) after treatment with FK866 for 48 h (5 nm MC38, 10 nm CT26) (*n* = 3). Scale bars = 10 µm; *< 0.05, **< 0.01, ***< 0.001.

To further evaluate the effects of inhibiting Nampt‐mediated NAD^+^ metabolism in the MC38 and CT26 CRC cell lines, different concentrations of FK866, an inhibitor of Nampt, were used on MC38 and CT26 cells for 24, 48, and 72 h; then, cell viability was measured by a CCK8 assay. FK866 reduced the viability of MC38 and CT26 cells in a concentration‐ and time‐dependent manner (**Figure** **3**A). The colony formation assay results were consistent with the CCK‐8 assay results, indicating that FK866 inhibited the viability of CRC cells (Figure 3A). Further transmission electron microscopy (TEM) revealed that cells treated with FK866 contained a smaller number of mitochondria, denser mitochondrial membranes and a decrease/disappearance of mitochondrial cristae, which are characteristics of ferroptosis (Figure 3B). Inspired by these results, we next measured the levels of intracellular ROS and lipid ROS, a hallmark of ferroptosis, by using the fluorescent probes DCFH‐DA and C11‐BODIPY. The results showed that intracellular ROS and lipid ROS in MC38 and CT26 cells were markedly elevated after treatment with FK866 (Figure 3C; Figure S1A, Supporting Information). In addition, the level of malondialdehyde (MDA), a product of lipid peroxides, also increased after treatment with FK866 (Figure 3D). Then, we detected the expression of several antioxidant genes, such as Gpx4, Nfe2l2 and Aifm2, which have been reported to be involved in ferroptosis.^[^ ^23^, ^24^, ^25^ ^]^ FK866 decreased the mRNA expressions of the Gpx4, Nfe2l2 and Aifm2 (Figure 3E). Collectively, these findings demonstrated that FK866 reduced cell viability and increased oxidative stress and lipid peroxidation in CRC cells, which strongly suggested that FK866 can induce ferroptosis in CRC cells.

### 2.3 Ferroptosis of CRC Cells Induced by FK866 Could be Rescued by a Ferroptosis Inhibitor

**Figure 4:**
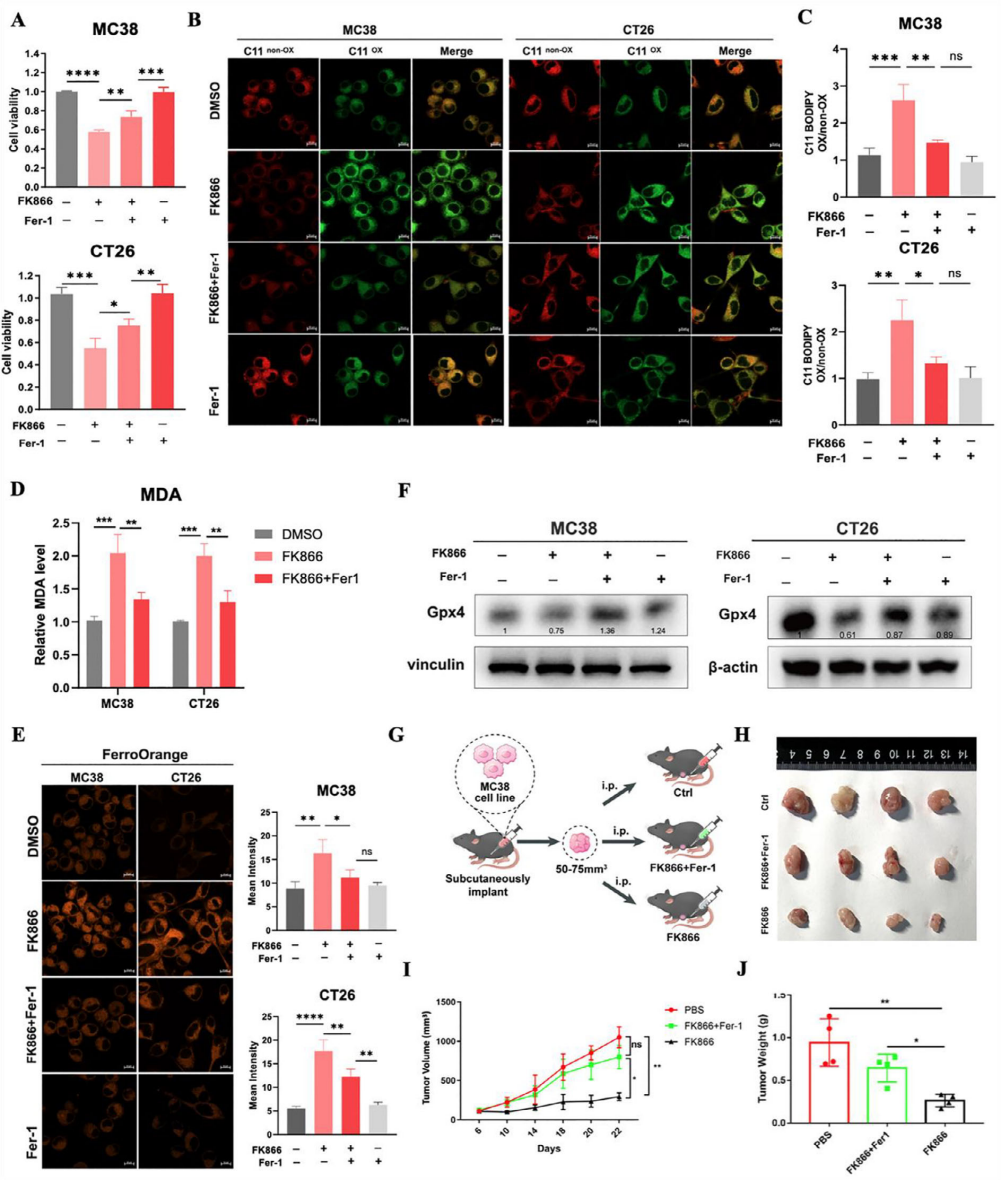
FK866 induces CRC cell death by triggering ferroptosis. A) Viability of MC38 and CT26 cells treated with FK866 (5 nm MC38 or 10 nm CT26) for 48 h in the absence or presence of Fer‐1 (5 µm) (*n* = 3). B,C) The relative lipid ROS levels of MC38 and CT26 cells were assayed via C11‐BODIPY fluorescence after treatment with FK866 (5 nm MC38 or 10 nm CT26) in the absence or presence of Fer‐1 (5 µm) (*n* = 3). D) The relative MDA levels of MC38 and CT26 cells after treatment with 5 nm MC38 or 10 nm CT26 in the absence or presence of Fer‐1 (5 µm) (*n* = 3). E) The relative iron levels of MC38 and CT26 cells were assayed via FerroOrange fluorescence after treatment with FK866 (5 nm MC38 or 10 nm CT26) in the absence or presence of Fer‐1 (5 µm) (*n* = 3). F) MC38 and CT26 cells were treated with FK866 in the absence or presence of Fer‐1 (5 µm) for 48 h, and Gpx4 protein expression was measured via western blotting. G) The scheme of the mouse experiments. MC38 cells were subcutaneously injected into C57BL/6 mice to establish a subcutaneous tumor model. Once the tumors reached an average volume of 50–75 mm^3^, the mice were randomly assigned to different treatment groups, with treatments administered every 3 days (*n* = 4). H) Representative tumor images from each treatment group (*n* = 4). I) Changes in tumor volume were monitored for each group (*n* = 4). J) The average weights of the tumors in each group (*n* = 4). Scale bars = 10 µm; *< 0.05, **< 0.01, ***< 0.001.

Next, the ferroptosis inhibitor Fer‐1 was used to further confirm that FK866 leads to ferroptosis in CRC cells. Our CCK‐8 results showed that Fer‐1 successfully reversed the cytotoxic effect of FK866 treatment on CRC cells (**Figure** **4**A). The increase in lipid peroxidation and MDA caused by FK866 was also reversed by Fer‐1 (Figure 4B–D). Iron dependence is another hallmark of ferroptosis, and we further measured iron levels using the FerroOrange assay. FK866 significantly increased the iron level, and Fer‐1 successfully reversed this change (Figure 4E). Further detection of ferroptosis markers revealed that FK866 decreased the expression of the ferroptosis suppressor Gpx4, and Fer‐1 rescued its expression (Figure 4F; Figure S2A,B, Supporting Information). Moreover, the effects caused by FK866 were confirmed in vivo and were partly reversed by the ferroptosis rescue agent Fer‐1 (Figure 4G–J; Figure S2C, Supporting Information). FK866 suppressed tumor growth in mice, and immunohistochemical staining of Ki67 and Gpx4 revealed that FK866 could inhibit cell proliferation and promote ferroptosis (Figure S2C, Supporting Information). Taken together, these findings strongly suggested that FK866 induced ferroptosis in CRC cells.

### 2.4 FK866‐Induced Ferroptosis Depends on the NAD+ Metabolism Pathway

**Figure 5:**
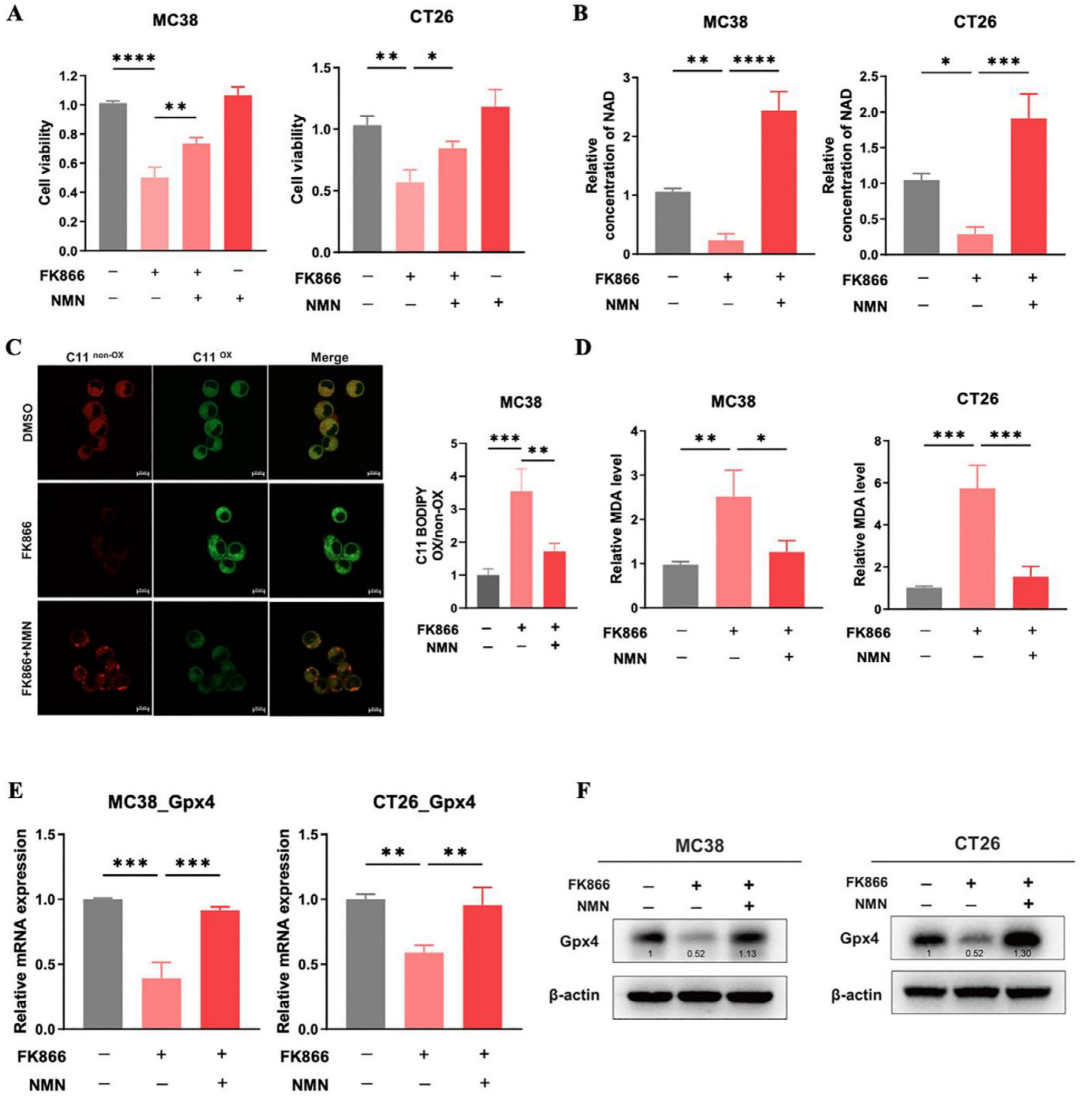
FK866‐induced ferroptosis depends on the NAD^+^ metabolism pathway. A) MC38 and CT26 cells were treated with FK866 (5 nm for MC38, 10 nm for CT26) and NMN (500 µm), and cell viability was analyzed by a CCK‐8 assay (*n* = 3). B) The NAD^+^ level was measured with or without FK866 (5 nm for MC38, 10 nm for CT26) and NMN (500 µm) (*n* = 3). C) Levels of lipid ROS detected with C11‐BODIPY 581/591 after treatment with FK866 (5 nm for MC38) and NMN (500 µm) (*n* = 3). D) The relative MDA level was detected with an MDA assay kit (*n* = 3). E) The mRNA levels of Gpx4 in MC38 and CT26 cells after treatment with FK866 (5 nm for MC38, 10 nm for CT26 and 500 µm NMN) for 48 h (*n* = 3). F) The protein levels of Gpx4 in MC38 and CT26 cells after treatment with FK866 (5 nm for MC38, 10 nm for CT26 and 500 µm NMN) for 48 h. Scale bars = 10 µm; *< 0.05, **< 0.01, ***< 0.001, ****< 0.0001.

As FK866 has been demonstrated to reduce NAD^+^ levels, it is unclear whether FK866‐induced ferroptosis is dependent on the NAD^+^ metabolism pathway. Therefore, we used NMN, a precursor of NAD^+^ synthesis, to counteract the inhibition of NAD^+^ by FK866 to explore the association between FK866‐induced ferroptosis and the NAD^+^ metabolism pathway. As shown in **Figure** **5**A,B, after treatment with FK866, the decreases in cell viability and NAD^+^ levels in MC38 and CT26 cells were significantly reversed by the addition of NMN. Furthermore, the increases in lipid ROS and MDA levels were also decreased by NMN supplementation (Figure 5C, D). In addition, the downregulation of Gpx4 caused by FK866 was completely reversed by NMN (Figure 5E,F). These results suggested that FK866‐induced ferroptosis depended on the NAD^+^ metabolism pathway.

### 2.5 FK866 Induces Ferroptosis via the Stat3‐Gpx4 Axis

**Figure 6:**
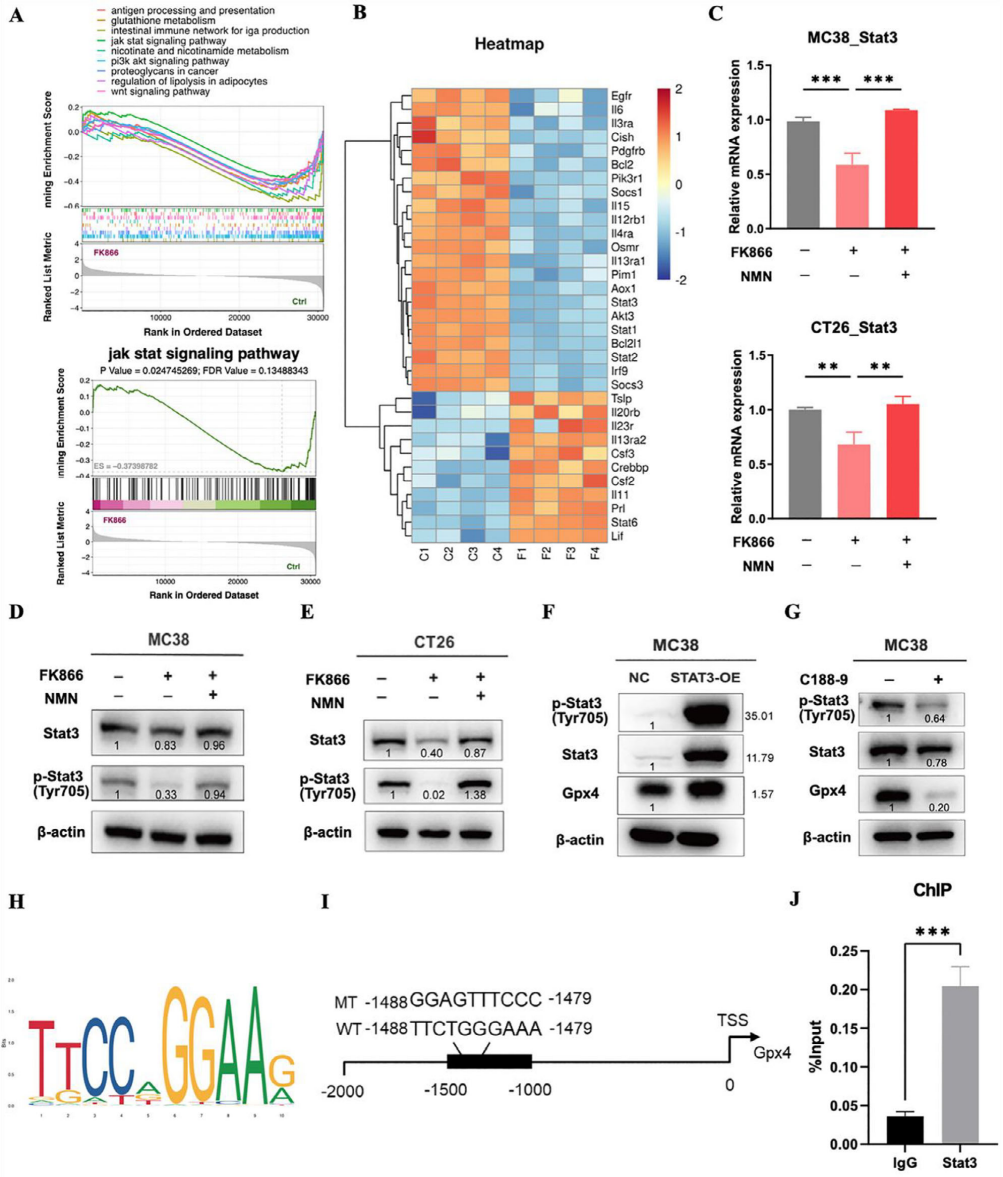
FK866 inhibits the Stat3‐Gpx4 signaling pathway. A) GSEA of differentially regulated pathways in MC38 cells treated with PBS or FK866 (*n* = 4). B) Differential expression of Jak‐Stat signaling pathway‐related genes (*n* = 4). C) The mRNA levels of Stat3 in MC38 and CT26 cells after treatment with FK866 (5 nm for MC38 or 10 nm for CT26) in the absence or presence of NMN (500 µm) for 48 h (*n* = 3). D,E) MC38 and CT26 cells were cultured with FK866 and NMN for 48 h. Whole‐cell lysates were subjected to immunoblotting with the indicated antibodies. F) The overexpression of Stat3 promoted the protein expression of Gpx4. G) Inhibition of the Stat3 signaling pathway by the Stat3 inhibitor C188‐9 decreased the protein expression level of Gpx4. H,I) A conserved Stat3‐binding motif was predicted by JASPAR, and schematic images of the potential Stat3 binding sites in the promoter of Gpx4 are shown. J) ChIP analysis of Stat3 occupancy at the Gpx4 promoter in MC38 cells. This experiment was conducted in triplicate. *< 0.05, **< 0.01, ***< 0.001.

To investigate the mechanism underlying ferroptosis in CRC cells upon FK866 treatment, we performed GSEA to compare the FK866 treatment group and the control group. GSEA revealed that one of the significantly enriched pathways was the Jak‐Stat signaling pathway (**Figure** **6**A), and we observed that the dysregulated genes included signal transducer and activator of transcription 3 (Stat3) (Figure 6B). Stat3 plays a critical role in tumorigenesis by promoting cell proliferation, survival, and immune evasion. The persistent activation of Stat3 is associated with cancer progression and resistance to therapy. However, whether Stat3 is involved in FK866‐induced ferroptosis in CRC remains elusive. Our results showed that FK866 treatment significantly inhibited the mRNA expression of Stat3, and the suppressed expression of Stat3 could be rescued by adding NMN (Figure 6C). In addition, the western blot results indicated that FK866 treatment significantly inhibited Stat3 expression and phosphorylation (Figure 6D,E). Importantly, NMN supplementation reversed the inhibition of Stat3 and phosphorylation of Stat3, suggesting that the downregulation of Stat3 caused by FK866 was dependent on NAD^+^ metabolism (Figure 6D,E). Next, we found that the overexpression of Stat3 significantly upregulated the protein expression of Gpx4 (Figure 6F). Moreover, the Stat3 inhibitor C188‐9 significantly decreased the expression of Gpx4 (Figure 6G). Then, we further analyzed how Stat3 regulates Gpx4 in CRC. The JASPAR website was used to predict the Stat3 binding sites in the Gpx4 promoter, and ChIP‒qPCR confirmed the presence of the Stat3 binding site in the Gpx4 promoter region (Figure 6H–J). Collectively, these data suggest that FK866 induces ferroptosis via the Stat3‐Gpx4 axis.

### 2.6 Combining FK866 with the Stat3 Inhibitor C188‐9 Leads to Synergistic Antitumor Effects In Vitro

**Figure 7:**
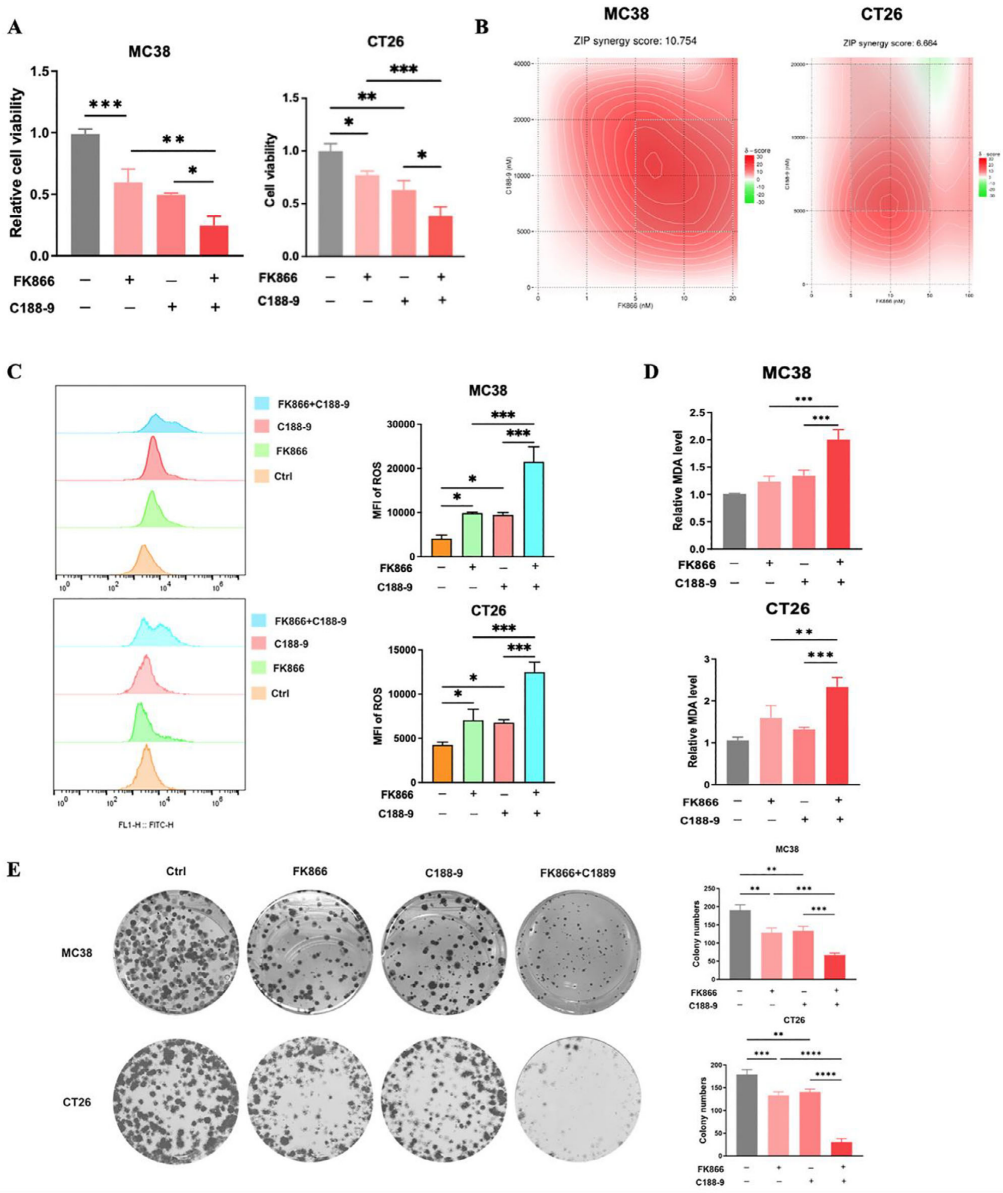
FK866 combined with C188‐9 exhibits synergistic antitumor effects. A) The viability of MC38 and CT26 cells treated with FK866, C188‐9, and FK866 + C188‐9 was measured by a CCK‐8 assay (the drug concentrations used for the MC38 cell line were 5 nm FK866 and 5 µm C188‐9, while for the CT26 cell line, they were 10 nm FK866 and 5 µM C188‐9) (*n* = 3). B) The drug combination matrix was constructed using SynergyFinder (version 3.0) (*n* = 3). C) ROS levels were detected by flow cytometry after cells were treated with FK866, C188‐9, or FK866 + C188‐9 (*n* = 3). D) MDA levels were detected by an MDA kit after cells were treated with FK866, C188‐9, or FK866 + C188‐9 (*n* = 3). E) Changes in the proliferative behavior of the cells were observed by colony formation assays after treatment with FK866, C188‐9, and FK866 + C188‐9 (MC38: FK866 5 nm, C188‐9 5 µm; CT26: FK866 10 nm, C188‐9 5 µm) (*n* = 3). *< 0.05, **< 0.01, ***< 0.001.

Since both the Nampt inhibitor FK866 and the Stat3 inhibitor C188‐9 can inhibit the Stat3‐Gpx4 signaling axis, we hypothesized that the antitumor effect on CRC could be synergistically enhanced by combining FK866 with C188‐9. As shown in **Figure** **7**A, the ability of FK866 and C188‐9 to inhibit cell viability was greater than that of any single drug. Further analysis of the drug combination matrix was performed using SynergyFinder (V3.0). The ZIP model showed that the interaction between FK866 and C188‐9 was synergistic in MC38 cells, but additive in CT26 cells (Figure 7B). In addition, compared to FK866 and C188‐9 alone, the two inhibitors combined and produced significantly more ROS and MDA in CRC cells (Figure 7C, D) and significantly inhibited cell proliferation (Figure 7E). These results indicate that FK866 combined with C188‐9 exhibits synergistic antitumor effects in vitro.

### 2.7 ROS‐Responsive Hydrogel‐Coated FK866 and C188‐9 Exhibit Synergistic Antitumor Effects and Induce Ferroptosis In Vitro

**Figure 8:**
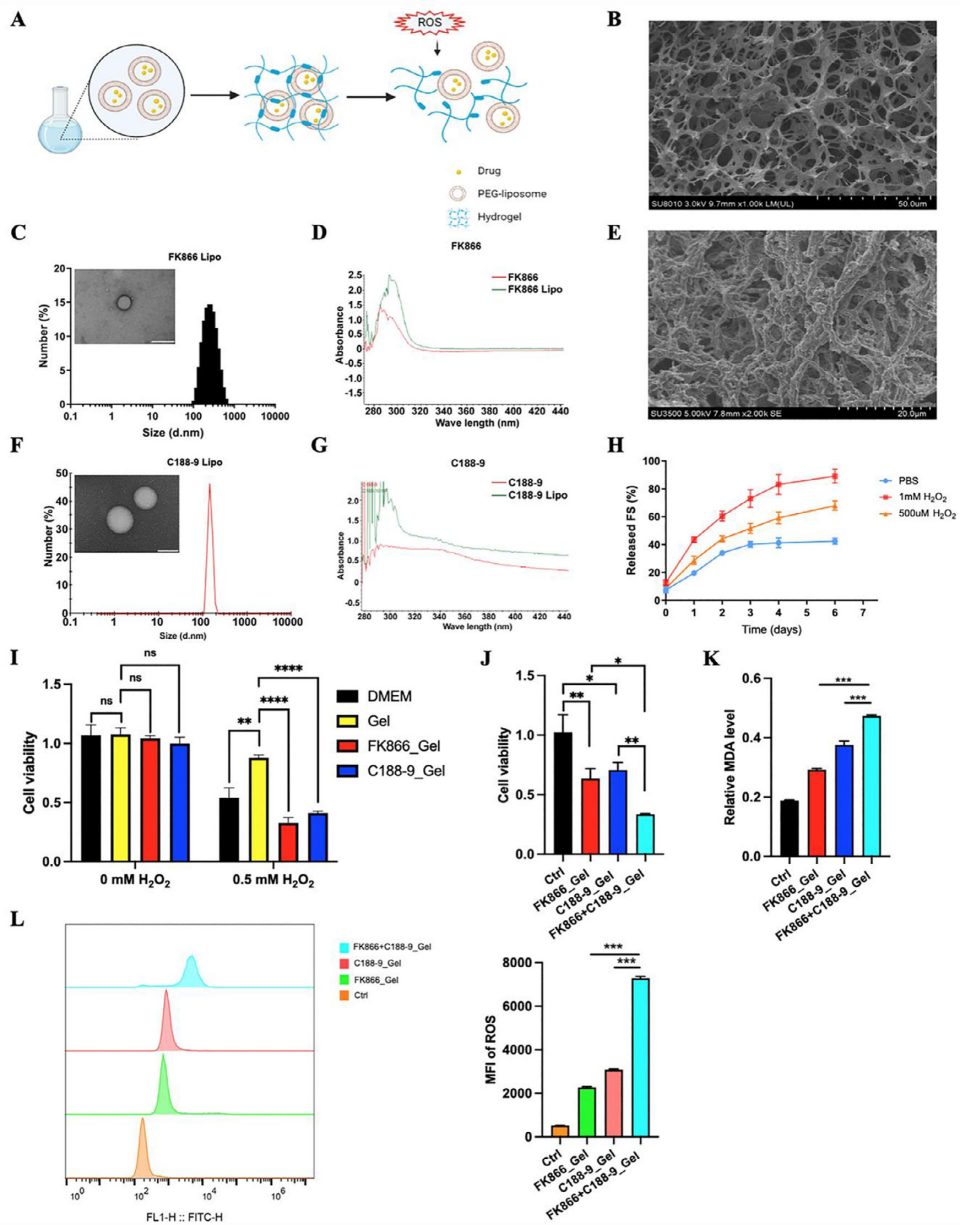
Preparation of ROS‐responsive hydrogels and in vitro anticancer and ferroptosis‐inducing effects of the hydrogels. A) Schematic illustration of the preparation of ROS‐responsive hydrogels loaded with drugs. B) SEM image of the ROS‐responsive hydrogel. Scale bar = 50 µm. This experiment was conducted in triplicate. C) The size distribution of liposomes loaded with FK866 and their morphology were observed by TEM (*n* = 3). Scale bar = 200 nm. D) Absorbance spectra of FK866 and liposome‐encapsulated FK866 (*n* = 3). E) SEM image of the ROS‐responsive hydrogel loaded with liposome‐encapsulated FK866 and C188‐9. Scale bar = 20 µm. This experiment was conducted in triplicate. F) The size distribution of liposomes loaded with C188‐9 and their morphology were observed by TEM (*n* = 3). Scale bar = 100 nm. G) Absorbance spectra of C188‐9 and liposome‐encapsulated C188‐9 (*n* = 3). H) Cumulative release profiles of fluorescein‐containing gels incubated in PBS without H_2_O_2_ or with different concentrations of H_2_O_2_ (n = 3). I) CCK‐8 assay for testing the drug release capacity of ROS‐responsive hydrogels incubated with or without H_2_O_2_ (*n* = 3). J) CCK‐8 assay for testing the killing effects of a hydrogel‐based strategy in which a single dose of FK866 (5 nm), C188‐9 (5 µm), or FK866 (5 nm) + C188‐9 (5 µm) was delivered upon H_2_O_2_ exposure (*n* = 3). K) The relative MDA levels of MC38 cells after different treatment (*n* = 3). L) ROS levels of MC38 cells were detected by flow cytometry after different treatment (*n* = 3). *< 0.05, **< 0.01, ***< 0.001.

As FK866 has shown high efficacy in preclinical trials as a monotherapy for many cancers, it has been evaluated by researchers in phase I and phase II clinical trials. But none of them were able to reach to phase III due to the severe systemic side effects of FK866. Recent research on NAMPT inhibitors has predominantly centered on synergistic drug combinations and precision‐targeted therapeutic strategies. As an emerging drug carrier, hydrogels can serve as in situ deposits for the sustained release of drugs, can reduce systemic adverse effects and are widely used for tumor drug delivery. Herein, we used a ROS‐responsive hydrogel to deliver FK866 and C188‐9. The ROS‐responsive hydrogel was constructed by crosslinking polyvinyl alcohol (PVA) with the ROS‐sensitive linker N¹‐(4‐boronobenzyl)‐N^3^‐(4‐boronophenyl)‐N^1^,N¹,N^3^,N^3^‐tetramethylpropane‐1,3‐diaminium (TSPBA). The liposome‐encapsulated drug was incorporated into ROS‐responsive hydrogels, which release the drug in response to elevated ROS levels to exert antitumor effects (**Figure** **8**A). Scanning electron microscopy (SEM) revealed the structure of the hydrogel scaffold (Figure 8B). FK866 and C188‐9 were initially encapsulated in polyethylene glycol (PEG) to prevent leakage from the hydrogels due to their small molecular size. Particle size distribution analysis revealed a uniform size distribution, with average diameters of 236.4 nm for FK866 and 123.4 nm for C188‐9 (Figure 8C,F). UV–vis spectroscopic analysis was performed to evaluate the optical properties of FK866 before and after liposomal encapsulation. The liposome‐encapsulated FK866 exhibited a noticeable spectral shift compared to its free form, suggesting that FK866 was effectively incorporated into the liposomal carrier (Figure 8D). A similar spectral shift was also observed for liposome‐encapsulated C188‐9 (Figure 8G). FK866_Gel was prepared by adding a PVA solution containing PEG‐FK866 to a TSPBA solution. C188‐9_Gel was prepared by incorporating a TSPBA solution containing PEG‐C188‐9 into a PVA solution. For FK866+C188‐9_Gel, a PVA solution containing PEG‐FK866 was mixed with a TSPBA solution containing PEG‐C188‐9. The morphology of FK866+C188‐9_Gel was examined by SEM, revealing that numerous particles were successfully loaded within the hydrogel matrix (Figure 8E). Next, we verified the ROS responsiveness by exposing the fluorescein containing hydrogel to different concentrations of H_2_O_2_ (Figure 8H). The fluorescein release profile indicated that the hydrogel degradation was controlled by the concentration of H_2_O_2_. In addition, the CCK‐8 assay results showed that the drug barely leaked in the presence of PEG‐encapsulated and hydrogel‐coated material after incubation in media without H_2_O_2_ exposure (Figures 8I). After 0.5 mm H_2_O_2_ exposure, both FK866_Gel and C188‐9_Gel exhibited anticancer effects (Figure 8I; Figure S3A,B, Supporting Information). More importantly, the FK866 + C188‐9_Gel treatment exhibited stronger inhibitory effects on CRC cells than those of single agent treatments (either the FK866_Gel or C188‐9_Gel treatment) (Figure 8J). Additionally, the FK866 + C188‐9_Gel treatment exhibited higher levels of MDA and ROS compared to the monotherapy groups, indicating that the combination therapy promotes synergistic induction of ferroptosis in tumor cells (Figure 8K,L). These results demonstrated that the ROS‐responsive hydrogel can efficiently load and release FK866 and C188‐9 for cancer treatment; in addition, administration of the hydrogel‐based combination of FK866 and C188‐9 exhibited stronger synergistic anticancer effects and more robust induction of ferroptosis in vitro than either agent alone.

### 2.8 ROS‐Responsive Hydrogel‐Coated FK866 and C188‐9 Significantly Inhibit CRC Tumor Growth with No Observable Adverse Effects

**Figure 9:**
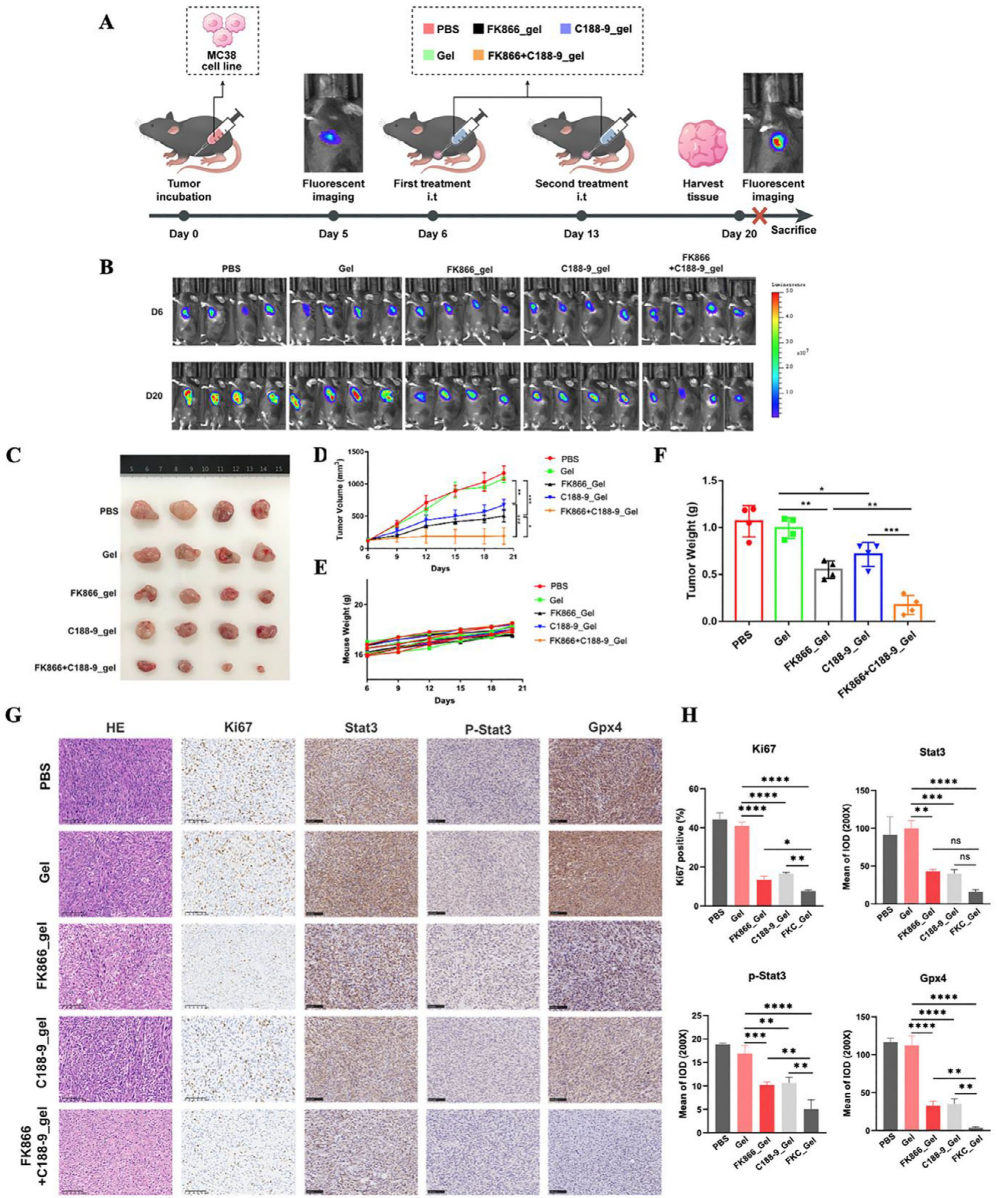
The ROS‐responsive hydrogels coated with FK866 and C188‐9 significantly inhibited CRC tumor growth. A) Treatment schedule for the MC38 cell‐treated tumor‐bearing mice (*n* = 4). B) In vivo bioluminescence images of MC38 cell tumor‐bearing mice after treatment with different formulations (*n* = 4). C) Representative images of the subcutaneous tumor model mice (*n* = 4). D) The changes in tumor volume were monitored and are shown for animals receiving different treatments (*n* = 4). E) The changes in mouse weight were monitored and are shown for animals receiving different treatments (*n* = 4). F) Average weights of the tumors after different treatments (*n* = 4). G,H) Representative IHC staining images and quantification of Ki67, Stat3, p‐Stat3, and Gpx4 protein expression in the indicated subcutaneous tumors (*n* = 4). *< 0.05, **< 0.01, ***< 0.001.

**Figure 10:**
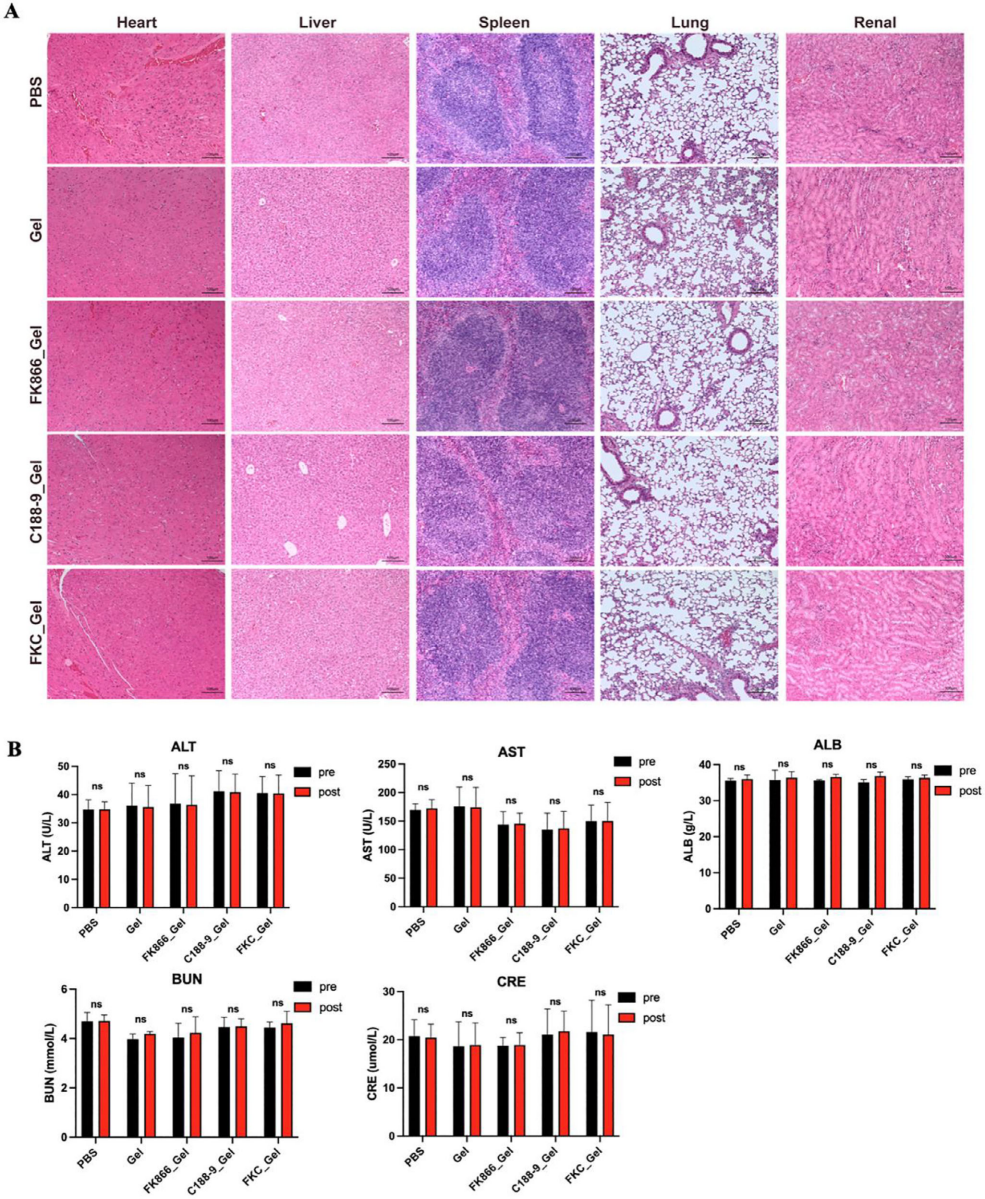
Evaluation of biocompatibility and adverse reaction in hydrogel‐based drug delivery. A) H&E staining of major organs including heart, liver, spleen, lung, and kidney, following hydrogel‐based drug delivery. Scale bar: 100 µm. FKC_ Gel: FK866+C188‐9_Gel (*n* = 4). B) Comparison of hepatic and renal function parameters before and after in vivo treatment (*n* = 4).

Encouraged by the strong synergistic effect achieved by combining FK866 and C188‐9 in vitro, we generated a subcutaneous colorectal tumor model in which mice were inoculated with MC38‐luc cells and treated with PBS, a blank hydrogel, or a therapeutic hydrogel containing FK866_Gel, C188‐9_Gel, or FK866+C188‐9_Gel (**Figure** **9**A). As shown in Figure 8B–F, the blank hydrogel did not affect the growth rate, while FK866_Gel, C188‐9_Gel, and FK866+C188‐9_Gel inhibited tumor growth (Figure 9B–F). The tumor volumes of the therapeutic groups were smaller, and the tumor weights were less than those of the control group. Notably, FK866+C188‐9_Gel exhibited better results than the individual drugs. More importantly, in mice treated with hydrogel, there was no significant decrease in body weight, suggesting that no significant adverse effects occurred (Figure 9E). The results of immunohistochemical staining for Ki67, Stat3, p‐Stat3 and Gpx4 were consistent with the above cell experiments, indicating that FK866+C188‐9_Gel treatment inhibited Stat3/Gpx4 signaling, activated ferroptosis and decreased tumor proliferation (Figure 9G,H). More importantly, no significant pathological changes were observed in major organs (heart, liver, lung, spleen, and kidney) following hydrogel‐based drug delivery treatment (**Figure** **10**A). Moreover, hepatic and renal function parameters remained within normal ranges before and after treatment, indicating no evident functional abnormalities (Figure 10B). These results indicated that local tumor therapy combined with FK866 and C188‐9 achieved a strong synergistic antitumor effect through inhibiting the Stat3/Gpx4 axis without causing significant adverse effects.

### 2.9 ROS‐Responsive Hydrogel‐Coated FK866 and C188‐9 Reshapes the Immune Microenvironment

**Figure 11:**
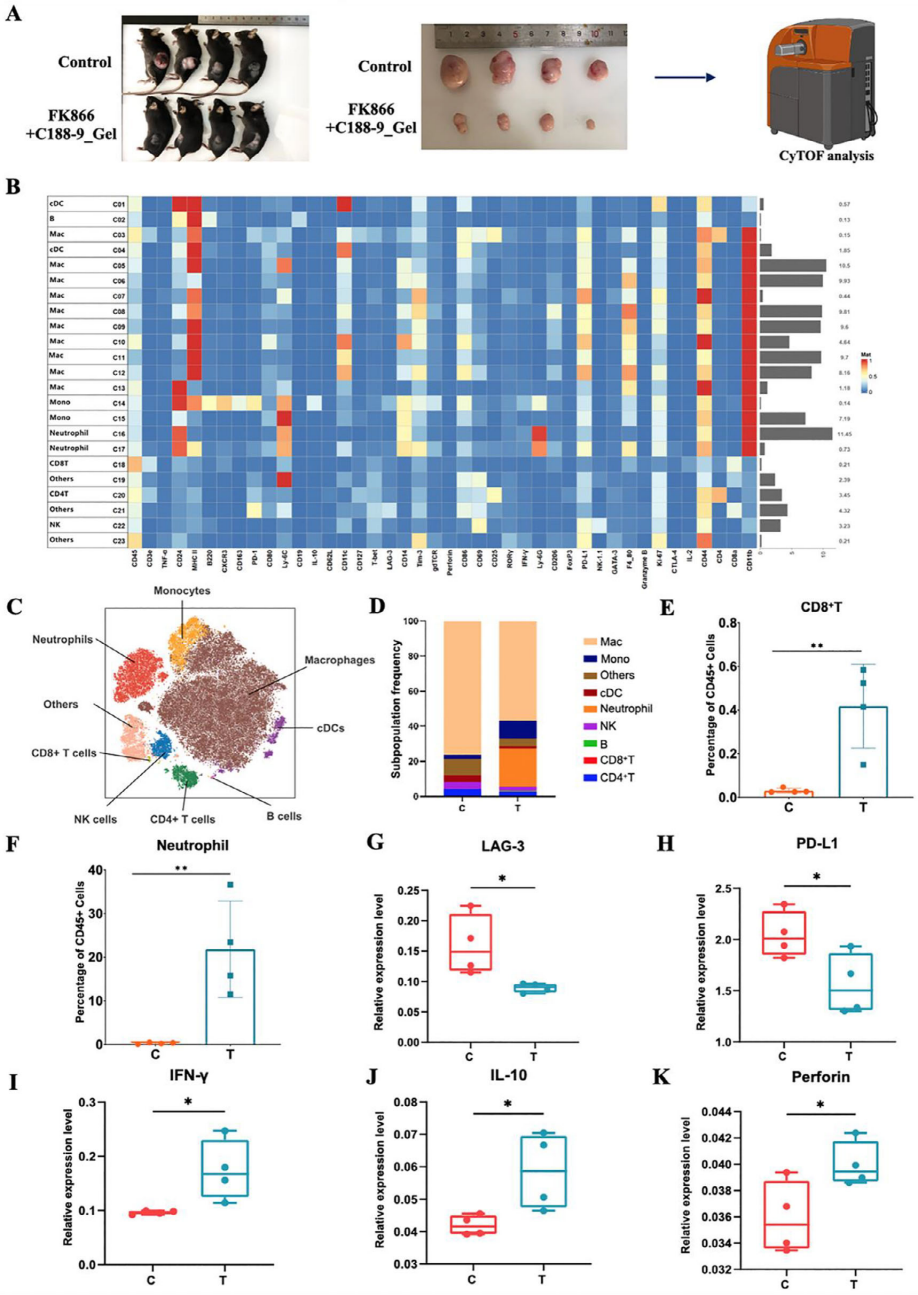
ROS‐responsive hydrogel‐coated FK866 and C188‐9 reshape the immune microenvironment. A) Schematic diagram of the preparation used for CyTOF analysis (*n* = 4). B) The project panel including 42 markers was used for CyTOF analysis. C) Nine major immune cell populations were confirmed by CyTOF analysis. D) Distribution of the immune cell clusters in the treated tumors (*n* = 4). E,F) Percentages of CD8^+^ T cells and neutrophils in the FK866+C188‐9_Gel‐treated and blank hydrogel‐treated groups (*n* = 4). G,H) Expression of immunosuppressive factors, including LAG3 and PD‐L1, in the FK866+C188‐9_Gel‐treated group and blank hydrogel‐treated group (*n* = 4). I–K) Expression of the functional markers IFN‐γ, IL‐10 and perforin in the FK866+C188‐9_Gel‐treated group and blank hydrogel‐treated group (*n* = 4). *< 0.05, **< 0.01, ***< 0.001.

Previous studies showed that Stat3 signaling or NAD^+^ metabolism can influence the function and recruitment of immunosuppressive cells within the tumor microenvironment.^[^ ^26^, ^27^ ^]^ However, the mechanism of FK866 and C188‐9 combination therapy against immunosuppressive microenvironment is unknown. The tumors treated with blank hydrogel (C group) or FK866+C188‐9_Gel (T group) were analyzed via mass cytometry by time of flight (CyTOF) (**Figure** **11**A). A project panel including 42 markers was applied to identify 23 clusters (Figure 11B), which were further confirmed as 9 major immune cell populations (Figure 11C). The immune patterns of the FK866+C188‐9_Gel‐treated tumors were different from those of the blank hydrogel‐treated tumors (Figure 11D). In the lymphatic system, there were significantly more CD8^+^ T cells in the FK866+C188‐9_Gel‐treated group than in the blank hydrogel‐treated group, which was consistent with the results obtained from the immunohistochemical staining of CD8 (Figure 11E; Figure S3C, Supporting Information). There was also a greater proportion of B cells in the FK866+C188‐9_Gel‐treated group than in the blank hydrogel‐treated group, but the difference was not statistically significant. There were fewer natural killer (NK) cells and CD4^+^ T cells in the FK866+C188‐9_Gel‐treated group than in the blank hydrogel‐treated group, but the differences were not significant. In the myeloid system, neutrophils and monocytes were increased, while dendritic cells (DCs) and macrophages were reduced in the FK866+C188‐9_Gel‐treated group compared to those in the blank hydrogel‐treated group. Notably, the differences in neutrophils between these two groups were statistically significant (Figure 11F). In addition, the levels of the functional markers IFN‐γ, IL‐10 and perforin were greater in the FK866+C188‐9_Gel‐treated group than in the blank hydrogel‐treated group, suggesting that immune cells were functionally activated after treatment with FK866 combined with C188‐9 (Figure 10I–K). Moreover, the levels of immunosuppressive factors, including LAG3 and PD‐L1, were lower in the FK866+C188‐9_Gel‐treated group than in the blank hydrogel‐treated group (Figure 11G,H), suggesting that FK866 combined with C188‐9 reshapes the immunosuppressive microenvironment. Taken together, these findings suggest that the combination of FK866 with C188‐9 affects CRC progression by regulating immune cell function.

## 3 Discussion

Despite advances in treatment, the prognosis for advanced colorectal cancer remains poor, underscoring the need for innovative strategies to improve patient outcomes. Recently, targeting NAD^+^ metabolism has gained attention as a potential approach for developing novel cancer therapies. In this study, we found that the Nampt inhibitor FK866 can induce ferroptosis through NAD^+^/Stat3/Gpx4 signaling in CRC. More importantly, we developed a ROS‐responsive hydrogel for the localized delivery of the Nampt inhibitor FK866 and the Stat3 inhibitor C188‐9, demonstrating potent synergistic anti‐cancer effects.

Recently, several novel forms of regulated cell death (RCD), including autophagy, ferroptosis, and pyroptosis, have been identified.^[^ ^28^ ^]^ Research from our group^[^ ^29^, ^30^, ^31^ ^]^ and others^[^ ^32^, ^33^, ^34^, ^35^ ^]^ has shown that these cell death mechanisms play significant roles in tumor initiation and progression. In this study, our RNA‐seq analysis revealed that FK866 contributes to the induction of ferroptosis in CRC. Ferroptosis is a form of RCD characterized by iron‐dependent lipid peroxidation. Abnormal iron metabolism, ROS accumulation and lipid peroxidation are the hallmarks of ferroptosis.^[^ ^36^ ^]^ But the role of NAD^+^ metabolism in regulating ferroptosis in tumor cells remains unclear. A recent study found that SIRT1‐mediated NAD^+^ consumption induces ferroptosis in gliomas.^[^ ^37^ ^]^ Conversely, in lung cancer, high doses of NMN were shown to induce ferroptosis, suggesting that elevated NAD^+^ levels can trigger this process.^[^ ^38^ ^]^ However, the NMN doses used in the lung cancer study were excessively high—100 to 10 000 times the standard dosage—raising concerns about the objectivity of the results and limiting their clinical relevance. In this study, we demonstrated that FK866 induces ferroptosis in CRC through an NAD^+^‐dependent mechanism, as evidenced by rescue experiments with the ferroptosis inhibitor Fer‐1 and NMN (downstream intermediate of Nampt).

Glutathione peroxidase 4 (Gpx4) is a key regulator of ferroptosis that interrupts lipid peroxidation by converting lipid hydroperoxides into non‐toxic lipid alcohols. Depletion of intracellular glutathione (GSH) and inactivation of Gpx4 result in cell ferroptosis.^[^ ^39^, ^40^ ^]^ Our RNA‐seq results revealed that FK866 modulates the key ferroptosis regulator Gpx4. However, it remains unclear how FK866 regulates Gpx4. Our RNA‐seq results suggested that FK866 is involved in regulating the Jak‐Stat signaling pathway, and further experimental results indicated that FK866 can regulate the total protein expression of Stat3 and the expression level of phosphorylated Stat3. By adding NMN, the downregulation of total protein expression of Stat3 and the phosphorylated Stat3 expression level due to FK866 can be rescued. Our results suggest that inhibitors of Nampt can suppress Stat3 expression in colorectal cancer cells via a mechanism mediated by NAD^+^. Furthermore, our ChIP assay results showed that Stat3 binds to the Gpx4 promoter region to promote its transcription. In summary, we propose for the first time that FK866 can induce ferroptosis in colorectal cancer cells through the NAD^+^ metabolism/Stat3/Gpx4 pathway.

Our study showed that FK866 can cause ferroptosis in colorectal cancer cells via the Stat3/Gpx4 axis, suggesting a potential therapeutic avenue for targeting the Stat3‐Gpx4 pathway. C188‐9 is a small molecular inhibitor of Stat3 that inhibits Stat3 signaling by preventing its dimerization and subsequent nuclear translocation, thereby blocking its transcriptional activity.^[^ ^41^, ^42^ ^]^ The inhibition of Stat3 signaling by C188‐9 further enhances the antitumor effect of FK866 by inhibiting the activation of prosurvival pathways downstream of Stat3. Our results revealed that FK866 combined with C188‐9 exhibits synergistic anticancer effects; compared with the individual agents, the combined agents more greatly inhibited cell viability and increased ROS, lipid ROS and MDA production in CRC cells. This is the first report on the remarkable therapeutic efficacy of FK866 and C188‐9 combinations in the treatment of colorectal cancer.

In addition, considering the results of previous studies in which FK866 did not enter phase III clinical trials due to its dose‐limiting toxicity, we designed an all‐in‐one hydrogel system in this study to simultaneously deliver FK866 and C188‐9 in situ. ROS‐responsive hydrogels are innovative cancer therapies designed to reduce high levels of ROS in the tumor microenvironment, allowing for targeted and controlled release of therapeutic agents directly within the tumor. This approach minimizes damage to healthy tissues and reduces systemic side effects, offering a promising strategy for effective and precise cancer treatment.^[^ ^43^, ^44^ ^]^ In our study, we constructed an ROS‐responsive hydrogel to load FK866 and C188‐9 in situ. Specifically, liposome‐wrapped FK866 and C188‐9 were encapsulated in ROS‐responsive hydrogels and found to exert remarkable therapeutic effects. This ROS‐responsive hydrogel, as a completely degradable platform, afforded sustained release of drugs and reduced systemic adverse effects and administration frequency.

Our previous study^[^ ^6^ ^]^ showed that FK866 inhibited the proliferation of CRC cells via the Wnt/β‐catenin pathway, and most studies on FK866 have focused only on tumor cells.^[^ ^11^, ^12^ ^]^ Recently, several researchers have elucidated the role of FK866 in modulating the tumor immune microenvironment. One key finding is its ability to enhance antitumor immune responses by promoting T‐cell infiltration into the tumor site.^[^ ^27^, ^45^ ^]^ In this study, we used CyTOF,^[^ ^46^ ^]^ a powerful mass cytometry technique for high‐dimensional single‐cell analysis, to investigate whether combination therapy reshapes the tumor immune microenvironment, thereby enhancing its synergistic anticancer effects. Additionally, alterations in the tumor immune microenvironment may offer valuable insights for subsequent combination immunotherapies, such as PD‐1/L1 inhibitors and LAG3 inhibitors. Our results demonstrated that combining FK866 and C188‐9 amplifies tumor inhibition by enhancing the anticancer immune response. This includes activating CD8^+^ T cells and neutrophils, as well as increasing the expression of key functional markers such as IFN‐γ, IL‐10, and perforin.

There are some limitations in our study and analysis. First, our study revealed that the Nampt inhibitor FK866 can induce ferroptosis in CRC; however, the anticancer effect of targeting NAD^+^ metabolism has been closely correlated with apoptosis and autophagy in other cancer types.^[^ ^47^, ^48^, ^49^ ^]^ FK866 may induce distinct forms of cell death in various subtypes of tumor cells. In the future, the single‐cell sequencing and other advanced techniques can be employed to elucidate the precise mechanisms underlying this phenomenon. Second, FK866 decreased the expression of Stat3 in an NAD^+^‐dependent manner, but the specific mechanism by which FK866 regulates the Stat3 signaling pathway needs to be confirmed by further experiments. Third, more studies are needed to clarify the regulatory effects and specific mechanism of FK866 and C188‐9 combinations in the immune microenvironment. Finally, further verification is required regarding the safety and efficacy of the combination of FK866 with C188‐9 therapy.

In summary, our study demonstrated a novel mechanism of FK866‐induced ferroptosis. FK866 inhibited the Stat3‐Gpx4 pathway through an NAD^+^‐dependent mechanism by impairing oxidative phosphorylation and inducing ferroptosis. We developed an ROS‐responsive hydrogel loaded with FK866 and C188‐9, which targets NAD^+^ metabolism and Stat3 signaling to enhance antitumor efficacy and reduce adverse effects. For the first time, this study analyzed the tumor immune microenvironment of colorectal cancer patients treated with FK866 combined with C188‐9 at the single‐cell level. Further research is still needed to fully elucidate the precise mechanisms underlying the synergistic effects of FK866 and C188‐9 and to optimize the dosing and delivery strategies. Nonetheless, the results are promising and very encouraging for further investigations in this cell metabolic based novel anti‐tumor treatment field.

## 4 Conclusions

Our study demonstrated that the Nampt inhibitor FK866 induces ferroptosis in CRC cells through the NAD^+^/Stat3/Gpx4 signaling pathway. To reduce systemic side effects of drugs, we developed a ROS‐responsive hydrogel for localized colorectal cancer treatment. Importantly, this hydrogel incorporates the NAD^+^ inhibitor FK866 and the Stat3 inhibitor C188‐9, which demonstrated synergistic antitumor effects.

## 5 Experimental Section

### Bioinformatics

TIMER2 (http://timer.comp‐genomics.org) was used to estimate the mRNA expression of Nampt in different tumors and adjacent normal tissues across all TCGA cancer types. UALCAN (http://ualcan.path.uab.edu/index.html) was used to explore the Nampt protein expression levels in primary CRC tumor and normal tissues based on the CPTAC dataset. The effect of the Nampt expression level on COAD patient survival was determined in UALCAN based on the TCGA dataset.

### Cell Culture and Treatment

Mouse MC38 and CT26 colon adenocarcinoma cells were maintained in the laboratory. MC38 and CT26 cells were cultured in Dulbecco's modified Eagle's medium (DMEM; Gibco; Thermo Fisher Scientific, USA) supplemented with 1% penicillin/streptomycin and 10% fetal bovine serum (FBS) at 37 °C in a 5% CO_2_ incubator. FK866 (S2799), C188‐9 (S8605), and ferrostatin‐1 (Fer‐1, S7243) were purchased from Selleck and dissolved in dimethyl sulfoxide (DMSO). β‐Nicotinamide mononucleotide (NMN, S5293) was dissolved in water.

### Cell Viability and Colony Formation Assays

Cell viability was determined using a cell counting kit‐8 (CCK8, Dojindo). A total of 3 × 10^3^ cells per well were seeded in 96‐well plates and treated with different concentrations of FK866 for 24, 48, or 72 h. Then, the cells were treated with 10% CCK8 reagent for 2 h, and the absorbance of each well was measured at 450 nm. For the colony formation assay, 1 × 10^3^ cells per well were seeded in 6‐well plates and treated with different concentrations of FK866 for 2 weeks. Then, the cells were washed with phosphate‐buffered saline (PBS), fixed with 4% paraformaldehyde for 10 min, stained with 0.5% crystal violet (Beyotime, China) for 15 min, washed with PBS and counted under a microscope. All experiments were conducted in triplicate.

### Transmission Electron Microscopy (TEM)

Cells were fixed with a solution containing 2.5% glutaraldehyde and 2% paraformaldehyde in 0.1 mol L⁻^1^ cacodylate buffer and then made into ultrathin sections. Digital images were obtained using a transmission electron microscope (HITACHI, Japan). All experiments were conducted in triplicate.

### NAD^+^/NADH Assay

NAD^+^ was measured with an NAD^+^/NADH kit (Beyotime, S0175) according to the manufacturer's instructions. A total of 1 × 10^6^ cells were prepared and digested. The concentration of total NAD^+^ (NAD^+^ + NADH) was measured by measuring the absorbance at 450 nm. All experiments were conducted in triplicate.

### Intracellular Iron and C11 BODIPY (581/591) Assay

Intracellular iron was detected with a Mito‐FerroGreen kit (DOJINDO, Kyushu, Japan) according to the manufacturer's protocol. The cells were incubated with a 1 mm C11‐BODIPY (581/591) probe (Invitrogen, D3861) for 30 min, washed with cold PBS three times and then visualized with a confocal microscope. All experiments were conducted in triplicate.

### Reactive Oxygen Species (ROS) Assay

For the ROS assay, after treatment with different drugs, the cells were treated with the fluorescent probe DCFH‐DA provided in the ROS Assay Kit (Beyotime, Shanghai, China) for 20 min, washed with PBS two times and detected via flow cytometry. All experiments were conducted in triplicate.

### Lipid Peroxidation Malondialdehyde (MDA) Assay

After treatment with different drugs, the collected cells were detected according to the instructions of the Lipid Peroxidation MDA Assay Kit (Beyotime, Shanghai, China). All experiments were conducted in triplicate.

### RNA Isolation and Relative Quantitative Real‐Time PCR (qRT–PCR)

Total RNA was extracted from different cells with Esunbio reagent according to the manufacturer's protocol. Complementary DNA was synthesized using reverse transcription kits (Yeasen, China). Quantitative real‐time PCR (qRT–PCR) was performed according to the manufacturer's instructions (Yeasen) with a Roche system. The 2−ΔΔCt method was used to analyze qRT–PCR data. The primers used were as follows: Actb‐F: GATGGTGGGAATGGGTCAGAAGG; Actb‐R: TTGTAGAAGGTGTGGTGCCAGATC; Gpx4‐F: ATAAGAACGGCTGCGTGGTGAAG; Gpx4‐R: TAGAGATAGCACGGCAGGTCCTTC; Stat3‐F: GCGGAGAAGCATTGTGAGTGAG; Stat3‐R: AGACGGTCCAGGCAGATGTTG; Nfe2l2‐F: CAGCCATGACTGATTTAAGCAG; Nfe2l2‐R: CAGCTGCTTGTTTTCGGTATTA; Aifm2‐F: CAGGAGTAGAGATGGCAGCAGAG; Aifm2‐R: CCGCACACAGGGCAGGAG.

### Western Blotting Assay

MC38 and CT26 cells were seeded in 6‐well plates, and different concentrations of FK866 were added after cell adhesion. The cells were washed with PBS and then lysed with RIPA buffer on ice for 30 min. Sodium dodecyl sulfate‒polyacrylamide gel electrophoresis (SDS‒PAGE) was used to electrophorese the total protein samples, which were subsequently transferred onto polyvinylidene fluoride (PVDF) membranes. The PVDF membranes were blocked with 5% BSA for 1 h and then incubated with the primary antibody at 4 °C overnight. The next day, the PVDF membranes were washed with Tris‐buffered saline with Tween (TBST), incubated with secondary antibodies at room temperature for 1 h and then washed with TBST three times. Finally, protein expression was detected with an enhanced chemiluminescence (ECL) kit. The antibodies used in this study were as follows: Gpx4 (1:1000, Abcam ab125066), Stat3 (1:1000, CST), phospho‐Stat3 (Tyr705) (1:1000, CST), β‐actin (1:1000, Huabio), SLC7A11 (1:1000, Proteintech), and vinculin (1:1000, Huabio).

### Preparation of Poly(ethylene glycol) (PEG) Liposomes

Five milligrams of DSP‐PEG‐COOH were dissolved in 10 mL of absolute ethanol, and the solution was placed on a dry magnetic mixer and stirred at medium speed. The drug (dissolved in 1 mL of absolute ethanol) was added to the liquid dropwise, and the mixture was stirred overnight. Afterward, the solvent was volatilized by spinning dry (60 °C, 110 rpm). The wall‐hung solute was rinsed off with 2 mL of PBS. Then, the size distribution of the PEG‐loaded liposomes was measured by dynamic light scattering (DLS, Zetasizer Nano), and the morphology was observed by TEM.

### Preparation of the ROS‐Responsive Hydrogel

N,N,N,N‐Tetramethylpropane‐1,3‐diamine (0.1 g, Aladdin) and 4‐(bromomethyl)phenylboronic acid (0.5 g, Aladdin) were dissolved in dimethylformamide (DMF, 10 mL, Sigma‒Aldrich) and stirred at 60 °C overnight. The next day, the mixture was washed with tetrahydrofuran (THF), filtered and dried under vacuum overnight. The purified TSPBA was weighed and dissolved in Milli‐Q water (10% TSPBA). PVA (72 kDa, 1 g, Aladdin) was added to Milli‐Q water (10 mL), followed by stirring at 90°C overnight. Hydrogel was formed by mixing the same volume of PVA (5%, w/w) matrix and a TSPBA linker (5%, w/w). The morphology of the hydrogel was detected by cryo‐SEM. Then, different concentrations of H_2_O_2_ (500 µm and 1 mm) were used to test the fluorescein cumulative release rate of the fluorescein‐loaded hydrogel to study the dynamics of hydrogel disassembly and drug release via an ultraviolet spectrophotometer. Next, the hydrogel‐loaded drugs were used for the CCK‐8 assay as described above to verify drug release.

### Subcutaneous Tumor Model

MC38 cells were resuspended in serum‐free medium at a density of 1 × 10^7^ cells/mL, and 100 µL of the cell suspension was injected subcutaneously into six‐week‐old C57BL/6 mice to establish a subcutaneous tumor model. When the tumors reached an average size of 50–75 mm^3^, the mice were randomly assigned to different treatment groups (FK866, 20 mg  kg⁻^1^ i.p., Fer‐1, 10 mg kg⁻^1^, i.p.) and were administered every 3 days. Tumors were harvested once they reached a volume of ≈1 000 mm^3^. The tumors were measured every 3 days, and the tumor volume was calculated by the following equation: V (mm^3^) = length × width^2^/2. Each group comprised four C57BL/6 mice. All mouse experiments were approved by the Animal Ethical and Welfare Committee of The Second Affiliated Hospital of Zhejiang University, School of Medicine (AIRB‐2021‐697), and were conducted in accordance with relevant ethical guidelines.

### Drug‐Loaded Hydrogel Treatment at the Tumor Site

MC38‐luc cells were used to better evaluate whether the baseline tumor burden was comparable across groups before different treatment, and to more accurately assess tumor viability and final tumor burden following drug administration. MC38‐luc cells were resuspended in serum‐free medium at a density of 1 × 10^7^ cells/mL, and 100 µL of the cell suspension was injected subcutaneously into six‐week‐old C57BL/6 mice to establish a subcutaneous tumor model. When the tumors reached an average size of 60–80 mm^3^, the mice were randomly assigned to different treatment groups. For local treatment, the following sterile insulin needles were prepared: one was loaded with TSPBA solution containing PEG‐FK866 (10 mg kg⁻^1^), one was loaded with PVA solution containing PEG‐C188‐9 (10 mg g⁻^1^), and the other two needles were loaded with only PVA solution and TSPBA solution. Then, FK866_Gel group was prepared by adding a PVA solution containing PEG‐FK866 to a TSPBA solution. C188‐9_Gel group was prepared by incorporating a TSPBA solution containing PEG‐C188‐9 into a PVA solution. FK866+C188‐9_Gel group was prepared by mixing a PVA solution containing PEG‐FK866 with a TSPBA solution containing PEG‐C188‐9. The hydrogel was immediately constructed when TSPBA and PVA were injected simultaneously into the tumor site. Treatments were applied every 7 days, and tumors were harvested when the volume reached ≈1 000 mm^3^. Each group comprised four C57BL/6 mice.

### Immunohistochemistry

Tumor tissues were fixed for 24 h in 10% formalin and paraffin embedded by Biossci Technology Co. (Wuhan, China). Immunohistochemical staining was performed according to the standard protocol. The following primary antibodies were used: GPX4 (Abcam, ab125066), STAT3 (CST, 9139), phospho‐STAT3 (Tyr705) (CST, 9145), Ki67 (CST, 12202), and CD8 (Abcam, 217344). Quantitative analysis was performed with Image‐Pro Plus Version 6.0 software.

### Bulk RNA‐Seq and Analysis

Bulk RNA‐seq was performed on MC38 cells following different treatments. KEGG pathway analysis, heatmap visualization, and GSEA were carried out using the LC OmicStudio platform. Each group has three replicates.

### Mass Cytometry Time‐of‐Flight (CyTOF) Analysis of the TME

The tumor tissues were digested, filtered, washed, and stained with a panel of 42 antibodies (Figure S4, Supporting Information). The signals were detected to evaluate the expression of the conjugated target molecule using the CyTOF system (Helios, Fluidigm, San Francisco, CA, USA). All CyTOF data were normalized and manually gated in FlowJo (version 10). Each group has four replicates.

### Statistical Analysis

All the results are presented as the means ± SDs and were analyzed with GraphPad Prism 9 software. Student's *t* test was used to compare differences between two groups, and one‐way ANOVA was used to compare differences among more than two groups. Statistical significance was defined as * *p* < 0.05, ** *p* < 0.01, and *** *p* < 0.001.

## Author Contributions

C.Y., M.M., and S.S. contributed equally to this work. C.Y., M.M., and S.S. share first authorship. Chenyang Ye, Mi Mi, Saimeng Shi, Ji Wang, and Ying Yuan conceived and designed the experiments. Chenyang Ye, Ji Wang, and Ying Yuan supervised the research. Chenyang Ye, Mi Mi, Saimeng Shi, Lina Qi, Shanshan Weng, Lu Wang, Yier Lu, Chao Chen, Yinuo Tan, Mengyuan Yang, and Cheng Guo performed the experiments. Chenyang Ye, Mi Mi, Saimeng Shi, Ji Wang, and Ying Yuan analyzed the data. Rui Bai and Xuefeng Fang provided suggestions and technical support. Chenyang Ye and Mi Mi wrote the manuscript. And all authors discussed the results and commented on it.

## Conflict of Interest

The authors declare no conflict of interest.

## Supporting information



Supporting Information

## Data Availability

The data that support the findings of this study are available in the supporting information of this article.
