# Supplementary material for: ROS‐Responsive Hydrogel for Localized Delivery of Nampt and Stat3 Inhibitors Exhibits Synergistic Antitumor Effects in Colorectal Cancer Through Ferroptosis Induction and Immune Microenvironment Remodeling
Source: Adv Sci (Weinh). 2025 Jun 10;12(33):e06599. doi: 10.1002/advs.202506599 (PMC12412517; doi:10.1002/advs.202506599)
Supplement: Supplementary file 1 — Supporting Information [file ADVS-12-e06599-s001.docx]

**ROS-Responsive Hydrogel for Localized Delivery of Nampt and Stat3 Inhibitors Exhibits Synergistic Antitumor Effects in Colorectal Cancer through Ferroptosis Induction and Immune Microenvironment Remodeling**

*Chenyang Ye ^#,*^, Mi Mi**^#^, Saimeng Shi^#^, Lina Qi, Shanshan Weng,* *Lu Wang, Yier Lu, Chao Chen, Yinuo Tan, Mengyuan Yang, Cheng Guo, Rui Bai, Xuefeng Fang, Ji Wang ^*^, Ying Yuan^*^*

**Figure S1. A.** ROS levels were detected by flow cytometry after cells were treated with FK866 (n=3). *<0.05, **<0.01, ***<0.001.

**Figure S2. A.** The mRNA expression of Gpx4 after treatment with different concentrations of FK866 for 48 h (n=3). **B.** Western blotting was used to detect the protein expression of Gpx4 after treatment with different concentrations of FK866 for 48 h. **C.** The levels of Ki67 and Gpx4 proteins in the indicated subcutaneous tumors were measured by IHC (n=4). *<0.05, **<0.01, ***<0.001.

**
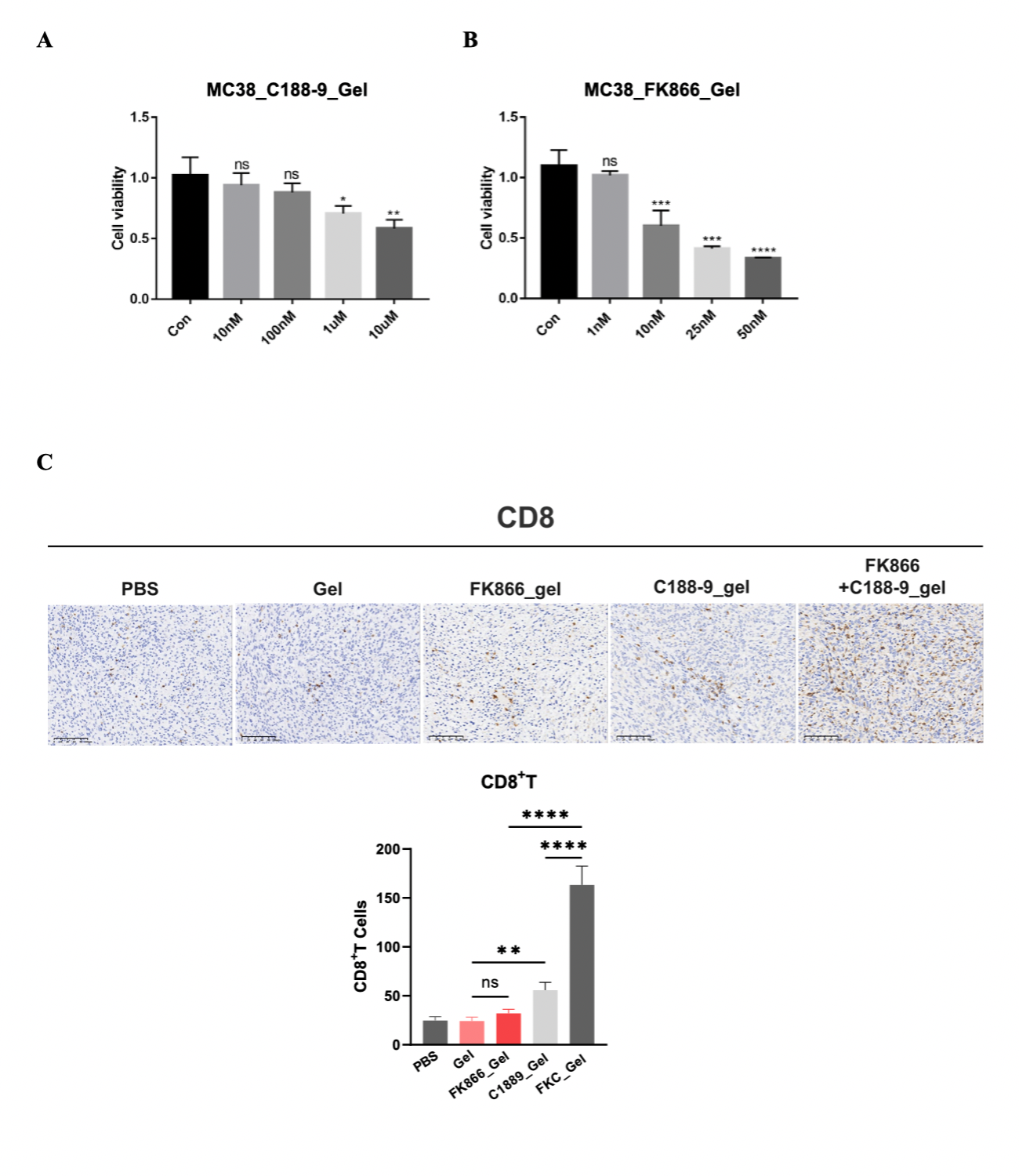
Figure S3.** **A.** MC38 cells were treated with different concentrations of C188-9_Gel for 48 h, after which cell viability was measured by a CCK-8 assay (n=3). **B.** MC38 cells were treated with different concentrations of FK866_Gel for 48 h, after which cell viability was measured by a CCK8 assay (n=3). **C.** The level of CD8 protein in the indicated subcutaneous tumors was determined by IHC (n=4). *<0.05, **<0.01, ***<0.001.

** Figure S4.** The CyTOF project panel of 42 markers.
